# Clinical Characteristics of Patients With Advanced Hepatocellular Carcinoma Who Transitioned to Subsequent Therapies Following Systemic Therapy

**DOI:** 10.1002/cam4.71616

**Published:** 2026-02-08

**Authors:** Kenji Imai, Koji Takai, Masashi Aiba, Shinji Unome, Takao Miwa, Tatsunori Hanai, Hiroyasu Sakai, Yohei Shirakami, Atsushi Suetsugu, Masahito Shimizu

**Affiliations:** ^1^ Department of Gastroenterology/Internal Medicine Gifu University Graduate School of Medicine Gifu Japan

**Keywords:** hepatocellular carcinoma, immune checkpoint inhibitor, overall survival, subsequent therapy, systemic therapy

## Abstract

**Aim:**

This study aimed to clarify the clinical characteristics of patients with advanced hepatocellular carcinoma (HCC) who transitioned to subsequent therapies following systemic therapy (ST).

**Methods:**

In total, 136 patients with unresectable HCC (26 hepatitis B, 47 hepatitis C and 63 others) receiving first‐line ST, including 31 patients treated with immune checkpoint inhibitors (ICIs), were enrolled. Clinical characteristics and adverse events observed during treatment, as well as overall survival (OS), progression‐free survival and post progression survival (PPS), were compared between patients who transitioned to subsequent therapies (2nd therapy group, *n* = 66) and those who did not (non‐2nd therapy group, *n* = 70).

**Results:**

Significant differences between the two groups were observed in OS (29.3 vs. 10.7 months, *p* < 0.001), PPS (11.3 vs. 2.9 months, *p* < 0.001), ALBI score (−2.48 vs. −2.34, *p* = 0.018), treatment with/without ICIs (24/42 vs. 7/63, *p* < 0.001), TNM stage (II/III/IVA/IVB; 15/26/7/18 vs. 3/27/10/30, *p* = 0.008) and the adverse events of appetite loss (*p* = 0.009) and proteinuria (*p* = 0.006). A favourable ALBI score (*p* = 0.005), treatment with ICIs (*p* = 0.002) and earlier TNM stage (*p* = 0.027) identified by logistic regression analysis and TNM stage II in men and prothrombin time ≥ 105% in women by classification tree analysis were found to be associated with a higher likelihood of transitioning to subsequent therapies.

**Conclusions:**

Initiating systemic therapy, including ICIs, before clinical stage progression and preserving the hepatic reserve is crucial for ensuring a smooth transition to subsequent therapies.

**Registry and the Registration No. of the Study/Trial:**

N/A.

## Graphical abstract

Among 136 patients with hepatocellular carcinoma (HCC) receiving first‐line systemic therapy (ST), 66 successfully transitioned to subsequent therapies. favourable ALBI scores, earlier TNM stage at the initiation of ST and treatment with immune checkpoint inhibitors (ICIs) were more likely to progress to subsequent therapies. Initiating systemic therapy, including ICIs, before clinical stage progression and preserving the hepatic reserve is crucial for ensuring a smooth transition to subsequent therapies.

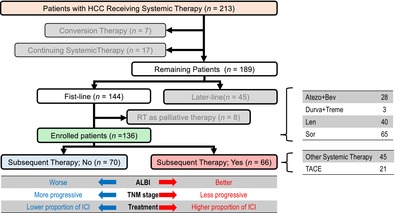

**Abbreviations**
AE: adverse event
AFP: α‐fetoprotein
Atezo: atezolizumab
Bev: bevacizumab
Cab: cabozantinib
CI: confidence interval
CR: complete response
CT: computed tomography
Durva: durvalumab
HCC: hepatocellular carcinoma
ICI: immune checkpoint inhibitor
Len: lenvatinib
OR: odds ratio
OS: overall survival
PD: progressive disease
PFS: progression‐free survival
PIVKA‐II: protein induced by vitamin K absence or antagonist‐II
PPS: post progression survival
PS: performance status
Ram: ramucirumab
Reg: regorafenib
SATI: subcutaneous adipose tissue index
SMI: skeletal muscle index
Sor: sorafenib
ST: systemic therapy
TACE: transcatheter arterial chemoembolization
Treme: tremelimumab
VATI: visceral adipose tissue index

## 1 Introduction

Hepatocellular carcinoma (HCC) is the most common primary liver cancer, with an estimated 957,000 new cases diagnosed worldwide and 830,200 deaths reported in 2020 [1, 2]. Systemic therapy (ST) is currently indicated in patients with unresectable HCC who are ineligible for local therapy [3]. STs for HCC can be broadly categorized into two groups [3]. The first group comprises immune checkpoint inhibitors (ICIs), which include programmed cell death ligand 1 inhibitors (atezolizumab [Atezo] and durvalumab [Durva]) and cytotoxic T‐lymphocyte‐associated protein 4 inhibitors (tremelimumab [Treme]). The second group includes anti‐angiogenic targeted therapies, such as sorafenib (Sor), lenvatinib (Len), regorafenib (Reg), cabozantinib (Cab), bevacizumab (Bev) and ramucirumab (Ram). According to the latest guidelines for HCC [3, 4, 5], STs with Atezo + Bev or Durva + Treme are the preferred first‐line options, while the others are considered later‐line therapies.

Significant advances in STs have greatly improved the prognosis of patients with unresectable HCC. However, in phase III clinical trials, the complete response (CR) rate for these STs alone has been reported to be remarkably low, ranging from 0% to 6.0%, while the percentage of patients who discontinued treatment due to disease progression, adverse events, or clinical deterioration during the trial was extremely high, ranging from 57.8% to 94.4% [6, 7, 8, 9, 10, 11, 12]. Thus, the effect of initial ST alone is inadequate, highlighting the importance of a prompt transition to subsequent therapies for improving treatment outcomes. Furthermore, while the prognosis of patients for whom subsequent therapy is considered unrealistic is expected to be extremely poor, the clinical characteristics facilitating a smooth transition to subsequent therapies in patients with advanced HCC receiving ST remain unclear.

This study aimed to identify the clinical factors associated with a successful transition to subsequent therapies by comparing patients with unresectable advanced HCC who received ST and transitioned to subsequent therapies with those who did not.

## 2 Methods

### 2.1 Patients and Treatment Strategy

**FIGURE 1:**
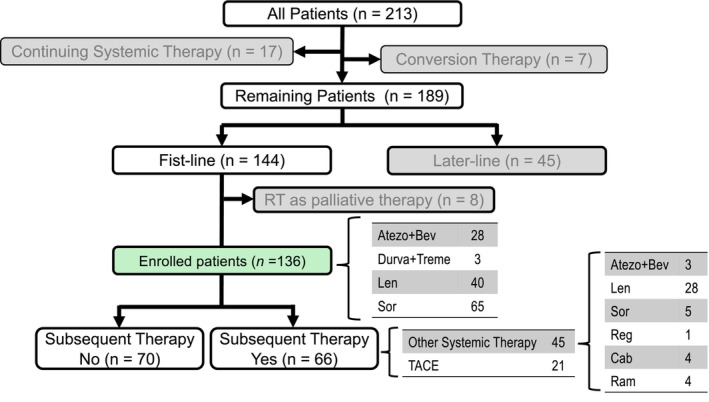
Patients flow in this study. Atezo, atezolizumab; Bev, bevacizumab; Cab, cabozantinib; Durva, durvalumab; Len, lenvatinib; Ram, ramucirumab; Reg, regorafenib; Sor, sorafenib; Treme, tremelimumab.

Between May 2009 and December 2023, 213 patients with HCC received ST at Gifu University Hospital. Of these, seven patients achieved CR after conversion therapy; 17 were still receiving ST at the end of the study period. Of the remaining 189 patients, first‐line ST was administered to 144 patients and later‐line to 45. Out of the 144 patients, this study included a total of 136 cases in which ST was initiated as first‐line therapy, excluding eight cases in which radiotherapy was subsequently added for palliative purposes. The patient flow chart for this study is presented in Figure 1. Drugs were selected based on the guidelines for ST of HCC [3, 4, 5]. Dynamic CT or MRI was performed at the initiation of ST and the therapeutic response was evaluated using these imaging modalities every 3–6 months based on physician judgment.

Patients enrolled in this study were informed about the study details and given the opportunity to withdraw their participation. The study design, including the informed consent procedure, was approved by the Ethics Committee of Gifu University School of Medicine on 13 February 2024 (Ethical Protocol Code: 2023–283).

### 2.2 Comparison of Cases That Transitioned to Subsequent Therapies and Those That Did Not

Among the enrolled patients, 66 transitioned to subsequent therapies (2nd therapy group) because of progressive disease (PD) (*n* = 33), adverse events (AEs) (*n* = 19), worsening hepatic reserve (*n* = 4), patient preference (*n* = 1), or others (*n* = 9), including 45 to other STs (Atezo+Bev/Len/Sor/Reg/Ram/Cab = 3/28/5/1/4/4) and 21 to transcatheter arterial chemoembolization (TACE) performed exclusively for salvage purposes rather than palliative intent. Subsequent therapy was chosen based on HCC clinical guidelines [3, 4, 5], similar to the selection of first‐line ST. The remaining 70 (non‐2nd therapy group) did not transition because of a decline in their general condition (*n* = 38), AEs (*n* = 17), worsening hepatic reserve (*n* = 9), or patient preference (*n* = 6). (Figure 1). All patients in the non‐2nd therapy group transitioned to best supportive care after discontinuation of first‐line ST. Baseline demographic and clinical characteristics of the enrolled patients at the time of ST initiation, AEs, treatment responses observed during treatment and clinical outcomes, including overall survival (OS) and progression‐free survival (PFS) and post progression survival (PPS) were compared between the two groups.

The skeletal muscle index (SMI; cm^2^/m^2^), subcutaneous adipose tissue index (SATI; cm^2^/m^2^) and visceral adipose tissue index (VATI; cm^2^/m^2^) were calculated as previously described [13]. AEs were graded according to the Common Terminology Criteria for Adverse Events (CTCAE), version 5.0. The treatment response was assessed according to the modified RECIST Criteria for Solid Tumors [14]. OS and PFS were defined as the interval from the date of initiation of ST to the date of death or PD, respectively. If these events did not occur, they were defined as the last observation day.

### 2.3 Statistical Analyses

Baseline demographic and clinical characteristics were compared between the 2nd and non‐2nd therapy groups using the Mann–Whitney U test for continuous variables and Fisher's exact test for categorical variables. Survival was estimated using the Kaplan–Meier method and differences between survival curves were assessed with the log‐rank test. Chronologic changes from baseline to end of treatment in SMI, SATI, VATI, α‐fetoprotein (AFP), protein induced by vitamin K absence or antagonist‐II (PIVKA‐II) and ALBI scores were analyzed using the Wilcoxon paired test. Odds ratios for transitioning to subsequent therapies were analyzed using logistic regression. Factors identified as significant in the univariate analysis were included in a multivariate analysis. In addition, classification tree analysis was performed to comprehensively examine clinical characteristics associated with higher transition rates to subsequent therapies, stratified by sex. All analyses were conducted using R software version 4.4.1, with the packages, ‘Rcmdr’, ‘RcmdrPlugin. EZR’, ‘rpart’, ‘rpart. plot’ and ‘ggplot2’ (R Foundation for Statistical Computing, Vienna, Austria; http://www.R‐project.org/ [accessed on 22 August 2024]).

## 3 Results

### 3.1 Baseline Clinical Characteristics and Treatment Course of the Enrolled Patients

For the 136 enrolled patients (108 men; median age 73 years), therapies included Atezo+Bev (28 patients), Durva+Treme (3 patients), Len (40 patients), Sor (65 patients). The median ALBI score was −2.43 and the performance status (PS: 0/1/2) was 105/22/9. The TNM staging (II/III/IVA/IVB) was 18/53/17/48 and the median AFP and PIVKA‐II levels were 96 ng/mL and 571 mAU/L, respectively. Prior treatment was received by 119 patients, while 83 underwent combination therapies during ST. The median treatment was 5.3 months (Table 1).

**TABLE 1:** Baseline demographic and clinical characteristics of the enrolled patients.

	All patients (*n* = 136)	2nd therapy group (*n* = 66)	Non‐2nd therapy group (*n* = 70)	*p*
Age (years)	73 [66, 78]	74 [66, 79]	72 [65, 78]	0.270
ECOG PS (0/1/2)	105/22/9	53/11/2	52/11/7	0.312
Sex (male/female)	108/28	49/17	59/11	0.203
Etiology (HBV/HCV/others)	26/47/63	15/21/30	11/26/33	0.574
BMI (kg/m^2^)	22.9 [20.4, 24.9]	22.9 [19.9, 25.0]	22.9 [20.9, 24.8]	0.678
SMI (cm^2^/m^2^)	43.0 [38.8, 49.1]	44.3 [38.7, 52.5]	42.1 [39.5, 47.0]	0.200
SATI (cm^2^/m^2^)	36.8 [24.1, 51.8]	37.3 [24.8, 52.6]	35.4 [22.7, 50.0]	0.596
VATI (cm^2^/m^2^)	43.0 [25.2, 59.6]	49.1 [31.9, 60.4]	39.8 [21.0, 57.3]	0.111
ALBI score	−2.43 [−2.77, −2.14]	−2.48 [−2.82, −2.25]	−2.34 [−2.74, −1.99]	0.018
Previous treatment (yes/no)	119/17	59/7	60/10	0.792
Combination treatment (yes/no)	83/63	23/43	30/40	0.382
1st line therapy (Atezo + Bev/Durva + Treme/Len/Sor)	28/3/40/65	21/3/21/21	7/0/19/44	< 0.001
1st line therapy (with ICI/without ICI)	31/105	24/42	7/63	< 0.001
Duration of 1st line therapy (month)	5.3 [1.8, 12.6]	5.9 [1.9, 13.0]	5.0 [1.8, 12.0]	0.664
TNM stage (II/III/IVA/IVB)	18/53/17/48	15/26/7/18	3/27/10/30	0.008
BCLC stage (A/B/C)	8/56/72	6/31/29	2/25/43	0.075
AFP (ng/mL)	96 [8, 1446]	52 [16, 1352]	176 [16, 1470]	0.149
PIVKA‐II (×10^3^ mAU/mL)	571 [56, 3111]	474 [58, 2357]	829 [59, 6842]	0.206
Best treatment response (CR/PR/SD/PD/NE)	6/24/45/56/5	4/16/22/23/1	2/8/23/33/4	0.230
Follow up period (month)	13.9 [5.8, 25.6]	18.4 [10.2, 30.3]	8.5 [4.1, 17.7]	< 0.001

*Note:* Continuous covariates are presented as median [interquartile range].

Abbreviations: AB, atezolizumab/bevacizumab; AFP, alpha‐fetoprotein; BCLC, barcelona clinic liver cancer; BMI, body mass index; CR, complete response; DT, tremelimumab/durvalumab; ECOG, eastern cooperative oncology group; HBV, hepatitis B virus; HCV, hepatitis C virus; ICI, immune checkpoint inhibitor; Len, lenvatinib; NE, not evaluable; PD, progressive disease; PIVKA‐II, protein induced by vitamin K absence or antagonist‐II; PR, partial response; PS, performance status; SATI, subcutaneous adipose tissue index; SD, stable disease; SMI, skeletal muscle index; Sor, sorafenib; VATI, visceral adipose tissue index.

### 3.2 Comparison Between the 2nd and Non‐2nd Therapy Groups at the Initiation of First‐Line Systemic Therapy

At the start of first‐line ST, significant differences were observed between the 2nd and non‐2nd therapy group in terms of ALBI score (−2.48 vs.−2.34, *p* = 0.018), first‐line ST (Atezo + Bev/Durva + Treme/Len/Sor/; 21/3/21/21 vs. 7/0/19/44, *p* < 0.001), first‐line ST (with ICI/without ICI; 24/42 vs. 7/63, *p* < 0.001) and TNM classification (II/III/IVA/IVB; 15/26/7/18 vs. 3/27/10/30, *p* = 0.008). No significant differences were noted in age, sex, liver condition, body composition, prior or combination therapy (Table 1).

### 3.3 Comparison of the Clinical Outcomes and Incidence of AEs Between the 2nd and Non‐2nd Therapy Groups

**FIGURE 2:**
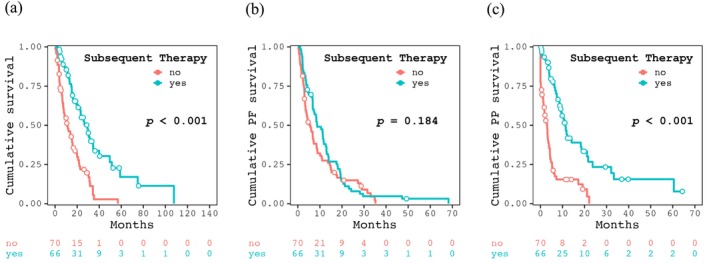
Kaplan–Meier curves for (a) cumulative survival, (b) cumulative progression‐free (PF) survival and (c) cumulative post progression (PP) survival between the patients who transitioned to subsequent therapy and those who did not.

Table 1 summarizes the clinical outcomes of patients in the 2nd and non‐2nd therapy groups. The median follow up period in this study was 13.9 [5.8, 25.6] months, with significant difference between the two groups (18.4 [10.2, 30.3] vs. 8.5 [4.1, 17.7] moths, *p* < 0.001). The median duration of first‐line ST was 5.3 [1.8, 12.6] months and was indistinguishable between the two groups (5.9 [1.9, 13.0] vs. 5.0 [1.8, 12.0] months, *p* = 0.664). However, the duration was significantly shorter in the ICI‐treated group than in the non‐ICI group (2.7 [1.4, 7.5] vs. 6.3 [2.3, 14.9] months, *p* = 0.006). The best responses of first line ST (CR/partial response [PR]/stable disease [SD]/PD/not evaluable [NE]), objective response and disease control rates were 4/16/22/23/1, 30.8% and 64.6% for the 2nd therapy group and 2/8/23/33/4, 15.2% and 50.0% for the non‐2nd therapy group. There was no significant difference between the two groups. In the 2nd therapy group, the median duration and the best therapeutic response (CR/PR/SD/PD/NE) in the 45 patients who received ST were 3.2 [1.2, 6.1] months and 1/4/20/18/2, respectively. The OS rates at 1, 2 and 3 years and the median OS were 81.9%, 55.0%, 33.7% and 29.3 months for the 2nd therapy group and 47.9%, 21.9%, 2.8% and 10.7 months for the non‐2nd therapy group (*p* < 0.001, Figure 2a). The PFS rates were 38.0%, 9.5%, 4.8% and 8.7 months for the 2nd therapy group and 14.9%, 2.2%, 0.0% and 5.6 months for the non‐2nd therapy group (*p* = 0.184, Figure 2b). The PPS rates were 44.2%, 23.4%, 15.6% and 11.3 months for the 2nd therapy group and 15.4%, 0.0%, 0.0% and 2.9 months for the non‐2nd therapy group (*p* < 0.001, Figure 2c).

The frequency and severity of AEs that occurred during ST are shown in Table 2. The incidence and severity of appetite loss and proteinuria were significantly higher in the non‐2nd therapy group (*p* = 0.009 and 0.006, respectively).

**TABLE 2:** Adverse events in response to systemic therapy.

	2nd therapy group (*n* = 66)	Non‐2nd therapy group (*n* = 70)	*p*
General fatigue (0/1/≥ 2)	40/12/14	34/8/28	0.058
Appetite loss (0/1/≥ 2)	42/9/15	28/9/33	0.009
Diarrhea (0/1/≥ 2)	50/13/3	53/10/7	0.411
Hypothyroidism (0/1/≥ 2)	52/5/9	64/2/4	0.119
Hand‐foot syndrome (0/1/≥ 2)	47/7/12	54/6/10	0.775
Proteinuria (0/1/≥ 2)	45/8/13	63/2/5	0.006
Hypertension (0/1/≥ 2)	40/2/24	52/2/16	0.233
Bleeding (0/1/≥ 2)	61/3/2	63/2/5	0.590

### 3.4 Chronological Changes in Body Composition, Liver Functional Reserve and Tumor Markers During Systemic Therapy

The chronological changes in body composition (SMI, SATI and VATI), liver functional reserve (ALBI score) and tumor markers (AFP and PIVKA‐II) during first‐line ST are shown in Table 3. AFP levels in the 2nd therapy group were the only parameter that did not exhibit a statistically significant change. In contrast, both groups demonstrated a significant decline in body composition and hepatic reserve and a considerable increase in tumor marker levels.

**TABLE 3:** Chronological changes in body compositions, liver functional reserve and tumor markers during systemic therapy.

	2nd therapy group (*n* = 66)			Non‐2nd therapy group (*n* = 70)		
	Introduction	End of treatment	*p*	Introduction	End of treatment	*p*
SMI (cm^2^/m^2^)	44.3 [38.7, 52.5]	41.8 [36.0, 50.2]	0.023	42.1 [39.5, 47.0]	39.6 [33.5, 43.1]	< 0.001
SATI (cm^2^/m^2^)	37.3 [24.8, 52.6]	30.7 [19.6, 48.0]	0.029	35.4 [22.7, 50.0]	35.0 [8.9, 43.7]	< 0.001
VATI (cm^2^/m^2^)	49.1 [31.9, 60.4]	40.7 [22.9, 57.6]	0.003	39.8 [21.0, 57.3]	30.8 [18.6, 53.2]	0.027
ALBI score	−2.48 [−2.82, −2.25]	−2.19 [−2.50, −1.82]	< 0.001	−2.35 [−2.75, −1.99]	−1.76 [−2.14, −1.25]	< 0.001
AFP (ng/mL)	52 [6, 1352]	126 [9, 1808]	0.053	176 [16, 1470]	532 [80, 8053]	0.005
PIVKA‐II (mAU/mL)	474 [58, 2357]	1459 [103, 11,248]	< 0.005	829 [59, 6842]	13,459 [753, 60,881]	< 0.001

Abbreviations: AFP, alpha‐fetoprotein; PIVKA‐II, protein induced by vitamin K absence or antagonist IISATI, subcutaneous adipose tissue index; SMI, skeletal muscle index; VATI, visceral adipose tissue index.

### 3.5 Clinical Characteristics of 2nd Therapy Group Determined by Logistic Regression and Classification Tree Analysis

Patients with favourable ALBI score (Odds ratio [OR] = 0.28, 95% confidence interval [CI]; 0.11–0.69, *p* = 0.005), treatment with ICIs (Atezo+Bev or Durva+Treme; OR = 4.65, 95% CI; 1.72–12.6, *p* = 0.002) and earlier TNM stage (*p* = 0.027) were found by logistic regression analysis to be more likely to transition to subsequent therapies (Table 4).

**TABLE 4:** Odds ratios for transitioning to subsequent therapy using logistic regression analysis.

	Univariate analysis		Multivariate analysis	
	Odds ratio (95% CI)	*p*	Odds ratio (95% CI)	*p*
Age (years)	0.98 (0.95–1.02)	0.325		
ECOG PS	0.68 (0.37–1.23)	0.199		
Sex (Female vs. Male)	0.54 (0.23–1.25)	0.151		
Etiology		0.56		
HBV	Reference			
HCV	0.59 (0.23–1.56)	0.289		
Others	0.67 (0.27–1.68)	0.389		
SMI (cm^2^/m^2^)	1.03 (0.99–1.07)	0.143		
SATI (cm^2^/m^2^)	1.00 (0.99–1.02)	0.477		
VATI (cm^2^/m^2^)	1.01 (0.99–1.03)	0.156		
ALBI score	0.35 (0.16–0.77)	0.009	0.28 (0.11–0.69)	0.005
Drug (with ICI vs. without ICI)	5.14 (2.03–13.0)	< 0.001	4.65 (1.72–12.6)	0.002
TNM stage		0.007		0.027
II	Reference		Reference	
III	0.19 (0.05–0.74)	0.017	0.20 (0.05–0.86)	0.031
IV (A or B)	0.12 (0.03–0.45)	0.002	0.14 (0.03–0.59)	0.007
AFP (ng/mL) (per 1000 unit)	0.98 (0.95–1.01)	0.253		
PIVKA‐II (mAU/mL) (per 1000 unit)	1.00 (0.99–1.00)	0.970		

Abbreviations: AFP, alpha‐fetoprotein; ECOG, eastern cooperative oncology group; HBV, hepatitis B virus; HCV, hepatitis C virus; ICI, immune checkpoint inhibitor; PIVKA‐II, protein induced by vitamin K absence or antagonist; IIPS, performance status; SATI, subcutaneous adipose tissue index; SMI, skeletal muscle index; VATI, visceral adipose tissue index.

**FIGURE 3:**
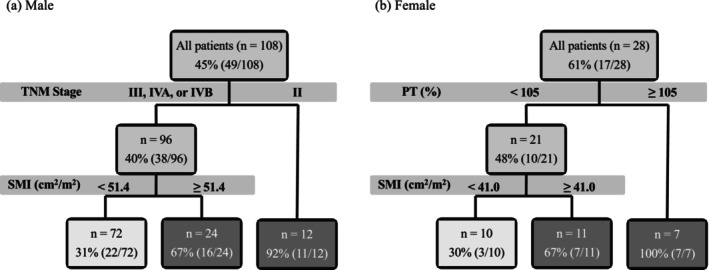
Classification tree analysis results for predicting patient eligibility for transitioning to subsequent therapy. Each node displays the total number of patients within the node and the proportion of those who progressed to subsequent therapy.

Classification tree analysis showed that 92% of men with TNM stage II were classified into the 2nd therapy group, whereas 31% of men with TNM stage III or higher and with an SMI < 51.4 cm^2^/m^2^ were classified into the 2nd therapy group. Additionally, 100% of women with a prothrombin time ≥ 105% were classified into the 2nd therapy group, whereas 30% of women with a prothrombin time < 105% and with an SMI < 41.0 cm^2^/m^2^ were classified into the 2nd therapy group. (Figure 3).

## 4 Discussion

This study demonstrated that patients with advanced HCC who exhibit favourable ALBI scores, earlier TNM stage at the initiation of ST and treatment with Atezo+Bev or Durva+Treme were more likely to progress to subsequent therapies. In addition, although PFS was not significantly different between the two groups, OS and PPS were significantly prolonged in the 2nd therapy group. These findings suggest that the extension of survival duration is primarily attributable to the impact of subsequent therapies rather than the efficacy of first‐line therapy. In this study, achieving CR after starting ST was extremely rare (7/213, 3%) in patients with advanced HCC and most patients (189/206, 92%) were forced to discontinue therapy as previously documented [6, 7, 8, 9, 10, 11, 12]. Therefore, to improve the survival of patients with advanced HCC, initiating ST, including ICIs, when the patient's liver functional reserve is favourable and the clinical cancer is at an earlier stage, ensuring a smoother transition to subsequent therapies, is recommended.

Tumor burden, liver function reserve and PS were included in the Barcelona Clinic Liver Cancer staging system for HCC [3, 5]. However, this study did not identify BCLC stage as a determinant influencing the transition to subsequent therapies. According to this algorithm, ST is indicated for patients at an intermediate stage (multinodular, preserved liver function reserve and PS of 0) and advanced stage (portal invasion and/or extrahepatic spread, preserved liver function reserve and PS of 1–2). As patients with intermediate‐stage disease are also recommended to undergo TACE [3, 5], there is often considerable debate as to whether TACE or ST should be administered to these patients. Considering the risk of liver function deterioration associated with TACE [15] and the results of this study, prioritizing ST in patients with intermediate‐stage HCC may be prudent. Recently, combination therapy with TACE and Len has attracted attention because of its high antitumor efficacy and favourable safety profile [16]. Furthermore, the abscopal effect, which refers to tumor regression outside the radiation field, induced by radiotherapy for advanced HCC may be further enhanced by immune checkpoint inhibitors [17]. Therefore, even after the initiation of ST, active consideration of the combination of TACE and radiotherapy may be beneficial for preserving the liver function reserves and maintaining high antitumor efficacy.

According to previous studies [18, 19, 20], the transition rate to second‐line ST varies widely, ranging from 1.7% to 54.5%, depending on the treatment era and the first‐line systemic agents used. Patients who permanently discontinued Sor had a particularly poor prognosis, with a median survival of only 4.1 months [21]. Our findings, in conjunction with previous research [18, 19, 20, 21], demonstrate that patients who are unable to transition to second‐line ST have a significantly shorter overall survival. This study also demonstrated that ST including ICIs appears to facilitate a more seamless progression to subsequent therapies than other treatment regimens. This may be related to ICIs having a higher antitumor efficacy than other regimens [6, 7]. In addition, Atezo+Bev treatment is reportedly associated with fewer AEs, including decreased appetite and general fatigue, than Len or Sor treatments [22, 23]. Preventing these AEs supports the maintenance of skeletal muscle mass and affects the smooth transition to subsequent therapies during treatment [22, 23]. In particular, as shown in this study, the preservation of skeletal muscle mass in men with TNM stage III or higher and in women with a prothrombin time < 105% is important for the transition to subsequent therapies. Since sarcopenia and rapid loss of skeletal muscle mass are critical prognostic factors in patients with HCC, it is important to consider their impact on muscle mass when choosing ST [24]. Therefore, patients with HCC treated with ICIs who have a low risk of muscle mass loss [22] are expected to have an easier transition to subsequent therapies because of their high antitumor efficacy and lower incidence of AEs that could lead to preservation of skeletal muscle mass. The latest HCC guidelines recommend Atezo + Bev or Durva + Treme as the first‐line therapy [3, 4, 5] and adherence to these guidelines is advisable from the perspective of facilitating a seamless transition to subsequent therapies.

This was a retrospective, single‐center study that had several limitations, including a relatively small sample size. In addition, because many of the treatment regimens evaluated in this study were newly introduced during the observation period, the timing of treatment initiation and agent selection may have evolved, potentially introducing bias into the results. While conducting survival analyses based on the presence or absence of subsequent therapies, there is a potential risk of introducing an immortal time bias. Owing to the inherent susceptibility of classification tree analysis to model instability, careful consideration is necessary when interpreting the results for female participants in this study, given the limited sample size. Prospective studies with larger patient cohorts are required to address these limitations.

In conclusion, among patients with advanced HCC, those with good ALBI scores, early TNM stage at the start of ST and those treated with Atezo + Bev or Durva + Treme were more likely to progress to subsequent therapies. Patients who progressed to subsequent therapies had significantly improved OS compared to those who did not progress; therefore, developing a treatment strategy that recognizes the appropriate progression to subsequent therapies is important.

## Author Contributions

**Kenji Imai:** conceptualization (lead), data curation (lead), formal analysis (lead), investigation (equal), writing – original draft (lead). **Koji Takai:** investigation (equal), supervision (lead), writing – original draft (supporting), writing – review and editing (equal). **Masashi Aiba:** investigation (equal), writing – original draft (supporting). **Shinji Unome:** investigation (equal), writing – original draft (supporting). **Takao Miwa:** investigation (equal), writing – original draft (supporting). **Tatsunori Hanai:** investigation (equal), writing – original draft (supporting). **Hiroyasu Sakai:** investigation (equal), writing – original draft (supporting). **Yohei Shirakami:** investigation (equal), writing – original draft (supporting). **Atsushi Suetsugu:** investigation (equal), writing – original draft (supporting). **Masahito Shimizu:** investigation (equal), supervision (lead), writing – original draft (supporting), writing – review and editing (lead).

## Funding

This research received no external funding.

## Ethics Statement

Patients enrolled in this study were given the opportunity to opt out with a full disclosure of study details. The study design, including the consent procedure, was approved by the Ethics Committee of Gifu University School of Medicine on February 13, 2024 (ethical protocol code: 2023–283).

## Conflicts of Interest

The authors declare no conflicts of interest.

## Data Availability

Data available on request from the authors.
